# Is the Thatcher Illusion Modulated by Face Familiarity? Evidence from an Eye Tracking Study

**DOI:** 10.1371/journal.pone.0163933

**Published:** 2016-10-24

**Authors:** Sandra Utz, Claus-Christian Carbon

**Affiliations:** 1 Department of General Psychology and Methodology, University of Bamberg, Bamberg, Germany; 2 Bamberg Graduate School of Affective and Cognitive Sciences (BaGrACS), Bamberg, Germany; Brock University, CANADA

## Abstract

Thompson (1980) first detected and described the Thatcher Illusion, where participants instantly perceive an upright face with inverted eyes and mouth as grotesque, but fail to do so when the same face is inverted. One prominent but controversial explanation is that the processing of configural information is disrupted in inverted faces. Studies investigating the Thatcher Illusion either used famous faces or non-famous faces. Highly familiar faces were often thought to be processed in a pronounced configural mode, so they seem ideal candidates to be tested in one Thatcher study against unfamiliar faces–but this has never been addressed so far. In our study, participants evaluated 16 famous and 16 non-famous faces for their grotesqueness. We tested whether familiarity (famous/non-famous faces) modulates reaction times, correctness of grotesqueness assessments (accuracy), and eye movement patterns for the factors orientation (upright/inverted) and Thatcherisation (Thatcherised/non-Thatcherised). On a behavioural level, familiarity effects were only observable via face inversion (higher accuracy and sensitivity for famous compared to non-famous faces) but not via Thatcherisation. Regarding eye movements, however, Thatcherisation influenced the scanning of famous and non-famous faces, for instance, in scanning the mouth region of the presented faces (higher number, duration and dwell time of fixations for famous compared to non-famous faces if Thatcherised). Altogether, famous faces seem to be processed in a more elaborate, more expertise-based way than non-famous faces, whereas non-famous, inverted faces seem to cause difficulties in accurate and sensitive processing. Results are further discussed in the face of existing studies of familiar vs. unfamiliar face processing.

## Introduction

Thompson (1980) first created and explicitly described the Thatcher Illusion. A Thatcherised face represents a manipulated photo of an upright face in which the eyes and mouth are inverted. Participants perceive this Thatcherised upright face as obviously grotesque, but have severe problems perceiving it as grotesque (or fail to do so) when the same face is inverted [1]. Therefore, an inverted Thatcherised face and a normal (non-Thatcherised) face are perceived in a similar way. One prominent (but also controversial) explanation is that the processing of configural information is disrupted in inverted faces, and therefore, the detection of relational differences is more difficult (e.g., [2–6]). Note that in this paper, “configural” processing refers to the encoding of so-called second-order relational information, i.e., distances between cardinal facial features, such as the eyes or between the nose and mouth (in line with [7–9]); for an overview of types of configural processing, see [10]. Hole and Bourne (2010; [9]) describe a reduction in sensitivity to spatial interrelationships between facial features and the rest of the face if faces are inverted. As the cardinal facial features (eyes and mouth) are uprightly orientated within an inverted Thatcherised face, the impression of a normal face arises [3], particularly when inspection time is limited. This would imply that featural and configural face information is differentially impaired through inversion: performance (accuracy and reaction times) deteriorates for faces differing in configural but not in featural information (e.g., [7; 11]). In face research literature, the Thatcher Illusion was often used for a prototypical illustration of powerful configural processing in the perception of upright, but not inverted faces. We would like to point to some specific findings of configural processing in Thatcher faces. Cornes et al. (2011) used faces and churches as stimuli and presented them in upright vs. inverted orientations, and with upright or inverted interior features (i.e., eyes, mouth, windows, doors), so-called “Thatcherised” stimuli [12]. Participants rated first the grotesqueness and identified afterwards the orientation of the stimuli and their features. The pattern of ratings was similar for both stimulus types, showing that increasing levels of Thatcherisation (only eyes or mouth vs. both regions) led to increasingly higher grotesqueness ratings in upright rather than inverted stimuli. Further analyses showed that the perceptual encoding was more sensitive to the distortions and the decision criterion was more liberal in upright compared to inverted faces, as well as inverted churches. However, there was substantial variation within the participant group. Finally, the authors could not find evidence for within-stimulus configurality, i.e. configural processing—revealed for example in Thatcherisation tasks—requires participants to compute and compare across stimuli rather than within stimuli. The authors saw a clear need for a critical view on configural processing in the Thatcher Illusion.

Further studies, however, could show that the entire process is more complicated than previously assumed, and that processing of configural information is selectively and not globally disrupted through inversion (e.g., [13]). In a recognition task using face stimuli in which featural and configural properties were independently manipulated in the eye or mouth regions, Tanaka et al. (2014) found disrupted perceptions of featural size and shape, and configural changes only in the mouth but not in the eye region in inverted faces [8]. In an extreme position, Rakover (1999), and Rakover and Teucher (1997) showed that inversion not only impairs configural processing, but also the processing of individual facial features, qualifying the configural processing hypothesis as only partially true [14; 15]. On the other hand, Yovel and Kanwisher (2004), for instance, could not find any differences between discriminating featural and configural information in inverted faces at all [16]. The investigation of eye movements in addition to reaction time or accuracy measures can possibly provide more information about the differences in processing configural and featural information in upright compared to inverted faces. Regarding differences in configural or featural processing of faces in general, participants in a study by Bombari, Mast and Lobmaier (2009) had to indicate if an intact test face matched the preceding scrambled, blurred or intact faces, while eye movements were measured [17]. Data revealed three modes of facial scanning: when cued with scrambled faces, participants showed a featural scan path with longer fixation times on individual features, i.e., a rather local type of detailed information search; when cued with blurred faces, a configural scan path with more inter-featural saccades was revealed, i.e., a more serial analysis of spatial relationships between features; if this information were available in the intact faces, participants showed a third scanning mode with most of the time fixating on the nose region (which was interpreted as a kind of “holistic” scanning), i.e., middle of the face with an assumed enlarged focus of attention to grasp the whole face without further eye movements. The holistic scan path shows simultaneous encoding across the broad regions of the face [17].

Examining the differences in eye movements between upright and inverted faces, it was repeatedly shown that participants attend, in upright faces, predominantly to the upper face region, particularly around the eyes (e.g., [18]). One plausible reason for this seemingly pre-fixed scanning behaviour is that most relevant facial information can be extracted from this region [19]. Williams and Henderson (2007) investigated the role of eye movements in producing the face inversion effect [20]. After ensuring that the used faces actually produce the face inversion effect, participants’ eye movements were recorded during learning faces and during a face recognition task. Results showed that the same facial features were fixated on during both tasks in both upright and inverted faces. This led the authors to conclude that face inversion effect is not evoked by different eye movement patterns [20]. In a response-contingent change detection paradigm, Xu and Tanaka (2013) again investigated eye movements to upright and inverted faces [21]. Participants had to indicate whether two sequentially presented faces of the same identity were the same or different. Contrary to Williams and Henderson (2007, [20]), they could demonstrate that participants showed more fixations on the nose and mouth regions for inverted faces, while the eyes and nose regions were the most fixated regions for upright faces. It seems, therefore, that inversion changed the eye movement behaviour in the mouth region. Interestingly, behavioural data showed that change detection was more difficult in inverted than in upright faces, especially when the mouth region was manipulated, although most fixations could be found for this region [21].

So far, the reported studies used “unfamiliar”, i.e., pre-experimentally unknown, faces to investigate the question of differences in processing upright or inverted faces. Thus, it was not revealed how the processing differs between familiar (here: famous faces, mostly facial depictions of celebrities) and pre-experimentally unfamiliar faces. From early face studies until now, it has been repeatedly shown that familiar faces are recognised faster and more accurately than unfamiliar faces (e.g., [22–24]). Highly familiar faces, such as famous faces, are often supposed to be particularly processed in a configural way (e.g., [25–27]; but see, e.g., [28; 29]) and are supposed to have stronger internal representations than unfamiliar faces [19]. Note, however, that here, highly familiar faces are not personally familiar faces for which we actually have deep expertise in processing [30]. Eye movement studies also reported marked differences between familiar and unfamiliar faces, e.g., upright famous faces were scanned faster compared to unfamiliar faces [19]. Heisz and Shore (2008) made participants familiar with novel faces through extensive exposure on, in sum, four consecutive days [31]. Participants had to perform a recall task each day, plus a recognition task on the last day; meanwhile, the experimenters captured the eye movements. The authors documented an increase in fixations in the region of the eyes and a concordant decrease of fixations in other regions (foremost in the nose and mouth regions) over the course of the experiment; this change of strategy in progressively focusing on the eyes region can be interpreted as an optimisation routine for capturing the most informative face regions, as this yields the best recall probabilities thereafter [31]. It seems, therefore, that the eye region might be very important to investigate and indicate the familiarity of faces.

Barton et al. (2006) investigated the effects of “expertise” (using upright and inverted faces) and “experience” (using famous vs. novel faces) on eye movement patterns using a face recognition task [19]. Expertise had an influence on eye movements, indicated by more fixations in the mouth and lower face region in inverted faces (i.e., where expertise is low) compared to upright faces (i.e., where expertise is high), where most fixations are in the eye region. The authors explained this effect with a loss in the rapid and efficient processing of spatial structure in inverted faces, therefore redirecting fixations to the lower part as an adaptation mechanism. Regarding the factor experience, upright famous faces were processed fastest and more fixations were made in the eyes and upper face region in novel compared to famous faces [19]. This finding is in contrast to Heisz and Shore’s (2008, [31]) findings, which revealed that fixations in the eye region increased with the familiarity of the faces. Barton et al. (2006) interpreted more scanning as a result of a higher need for more information, which was necessary for novel upright faces. Therefore, expertise shown for upright faces was revealed by less scanning for decision in the lower half of the face, and experience was revealed by faster decisions and less need for scanning the eyes region in famous faces compared to novel faces [19]. This study revealed the importance of investigating eye movements not only in the mouth but also in the eye region to get a clear picture of familiarity effects in face processing. Althoff and Cohen (1999) investigated the influence of prior knowledge on the eye movements of famous and non-famous faces, more specifically if differences in the pattern are due to the nature of the task [18]. To prime participants’ mental representations of the famous faces, participants had to rate names according to familiarity and likelihood of recognition. In the actual experiment, after the presentation of faces for 5 seconds, participants had to judge faces regarding fame or expressed emotions. Results showed significantly more fixations in the eye region and less in the mouth region of famous faces compared to non-famous faces (in accordance with [31]), modulated by the type of task, i.e., overall less time spent on the mouths of famous faces during the fame judging task compared to non-famous faces. In the fame judging task—even within 2 seconds of viewing—more fixations were made, more regions were sampled, and more constraints in the transitions among consecutive fixation locations (similar to the emotion judging task) was observed for non-famous faces, necessary for extracting as much information as possible from novel faces. Overall, prior processing can affect face processing, irrespective of the task [18]. Influences on eye movements in the eye and mouth region were again demonstrated and highly important to be investigated.

Interestingly, although the Thatcher Illusion addresses aspects of configural processing, studies on this phenomenon never employed both familiar and unfamiliar within one design. Instead, studies investigating the Thatcher Illusion *either* used famous faces, i.e., actors, supermodels, TV stars, royals (e.g., [3–5; 32], not to forget the initial demonstration with Margaret Thatcher, the former and widely well-known British Prime Minister in [1] *or* non-famous faces, e.g., [33–37]). Consequently, due to the missing direct comparison of both conditions, we do not know how “familiarity” actually impacts the perception of the Thatcher Illusion, not only behaviourally, but also in terms of eye movements. Since some studies are showing that facial identity and expression are probably processed along parallel processing streams (e.g., [38; 39]), it is also interesting to see if this is actually the case, i.e., that familiarity and Thatcherisation are not influencing each other.

In the present study, participants had to evaluate famous and non-famous faces according to their grotesqueness (similar to the basic procedure in [32]). To ensure that the faces from our set of famous faces were indeed recognised as images depicting famous faces known to our sample of participants, participants in a pre-study rated supposedly famous faces according to their actual familiarity. The main experiment tested how familiarity (famous/non-famous) of the presented faces influences the perception of Thatcherised faces when taking reaction times (RTs) and accuracy of grotesqueness ratings into account. Additionally, we recorded eye movements, since they were repeatedly shown to contain important information regarding face processing and analysed if familiarity has an impact on them. We expect differences in eye movements in general and more specifically to the eyes and mouth region depending on familiarity of the presented faces and on the orientation of the faces (upright or inverted). More specifically, we expect overall shorter fixation duration and dwell time, and fewer number of fixations for upright famous faces compared to non-famous faces [18; 19]; for unfamiliar and famous faces, we expect upright faces to be scanned predominantly (higher number of fixations) around the eyes and inverted faces predominantly (higher number of fixations) around the mouth (e.g., [18, 19 & 21]); however, while no differences between upright and inverted faces could be revealed, [20], for famous faces, there is a stronger focus on the eyes compared to unfamiliar faces [31]. However, it is also possible to find a smaller focus on the eye region for famous faces, because there might be less need for scanning this region in famous compared to novel faces [19].

Furthermore, RTs and accuracy should also reveal differences in the processing of famous and non-famous faces, since it was shown that participants are more accurate and faster in processing famous faces. Since expertise with a face (which is the case for famous faces) should theoretically increase configural processing, we expect faster and more accurate processing for famous, upright, non-Thatcherised faces, compared to non-famous faces and a bigger inversion effect for famous faces, i.e., larger difference in performance between upright and inverted faces if they are famous. It has been shown that–with some difficulty–the identity of a Thatcherised face can be recognized when the face is inverted, whereas the ability to perceive the grotesqueness of facial expression is nearly fully lost (see Table 1 in [4]). This dissociation is in line with some studies showing that facial identity and expression are probably processed along parallel processing streams (e.g., [38; 39]). Therefore, familiarity should not influence the perception of the Thatcher Illusion, and result in no interaction between the factors of Thatcherisation and familiarity following these sources that claim independent processing of identity and expression, following the legacy of the functional model of face processing initially created by Bruce and Young in 1986 [25]. Note however, that later studies could show some dependencies between processing of identity and expression [40, 41].

## Method

### Participants

**Fig 1:**
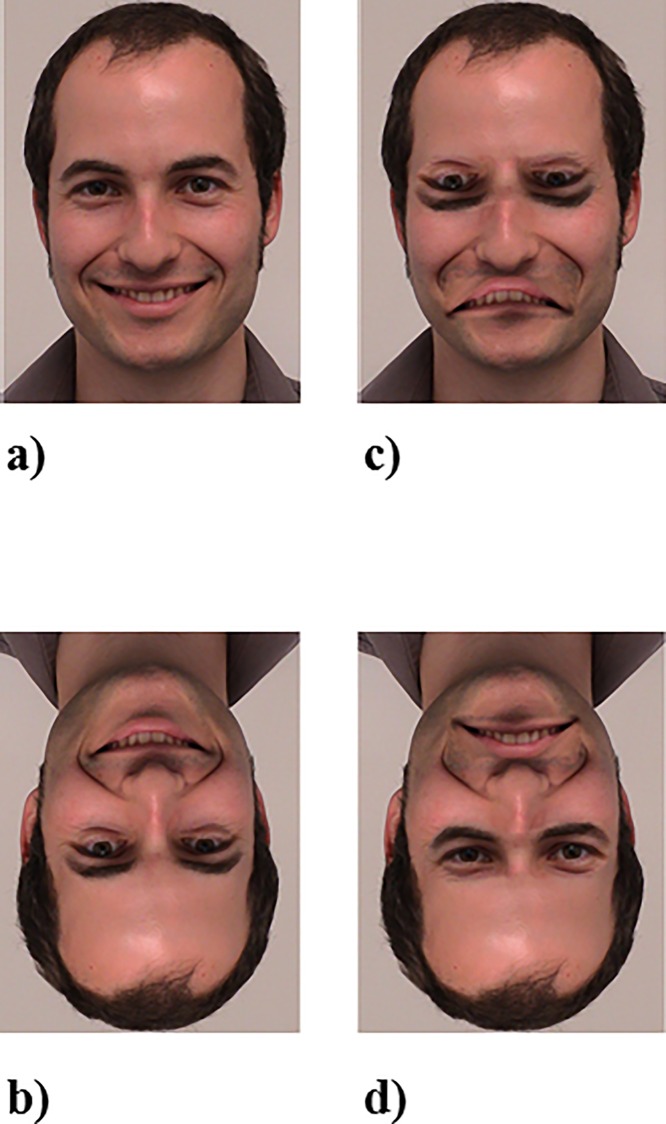
Example stimuli. Example for a male, non-famous face: a) upright, non-Thatcherised; b) inverted, non-Thatcherised; c) upright, Thatcherised; d) inverted, Thatcherised.

**Fig 2:**
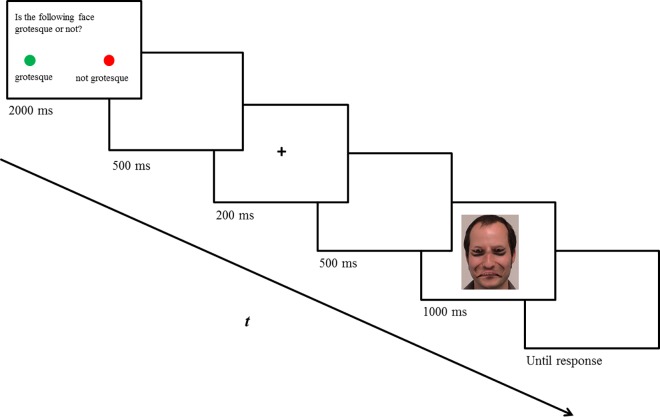
Trial schema. Presentation of a non-famous, upright, and Thatcherised face.

Twenty-six participants (23 female) participated with ages ranging from 19 to 31 years (*M*_*age*_ = 21.5 yrs; *SD*_*age*_ = 3.1). All participants had normal or corrected-to-normal vision (assessed by a standard Snellen eye chart test) and normal colour vision (assessed by a short version of Ishihara colour test). Participants were undergraduates from the University of Bamberg who received course credit points for their participation. They had no prior experience with the present task and were naïve to the purpose of this experiment, except for three participants, who were excluded from the data analysis, leading to a final number of 23 participants (20 female). The study was conducted according to the principles expressed in the Declaration of Helsinki and according to ethical principles of the German Psychological Society and the Association of German Professional Psychologists. Each participant was made aware of their right to withdraw themselves and their data from the study without consequences and without giving reasons. Written informed consent was given by each participant. The ethics committee of the University of Bamberg classified the study as ethically unproblematic and approved the study. The details and the rationale of the study were discussed with every participant on completion of the experiment. The individual pictured in Figs 1 and 2 in the present manuscript has given written informed consent (as outlined in PLOS consent form) to publish his picture.

### Apparatus

Participants were seated approximately 55 cm in front of a 23-inch Samsung Syncmaster 2233 TFT monitor running at a 1,680 × 1,050 pixel screen resolution with a refresh rate of 60 Hz controlled by a Thermaltake LanboxLite PC. Participants responded with two clearly marked keys on a RESPONSEPixx (VP-BB1) button box (accuracy ≤ 1 ms, manufactured by VPixx Technologies Inc., Canada). To ensure that the participants were not moving their heads, a chin rest was adjusted at a comfortable height (50 cm away from the plane of the monitor). Eye movements were measured using the EyeLink 1000 system (sample rate of 1,000 Hz; spatial accuracy of 0.25°‒ 0.5°, manufactured by SR Research Ltd., Canada). While the viewing was binocular, the recording was monocular, measuring right eye movements only, as this is a standard procedure in eye-tracking studies (e.g., [42]). Stimuli, trials and experimental blocks were created with the up-to-date SR Research Experiment Builder 1.10.1025 ensuring high precision in executing the correct timing of the study.

### Stimuli and Materials

A total of 128 facial stimuli were used, consisting of eight face exemplars x 2 gender (female/male) x 2 orientation (upright/inverted) x 2 Thatcherisation (Thatcherised/non-Thatcherised) x 2 familiarity (famous/non-famous). One example for a non-famous male face can be extracted from Fig 1. Non-famous faces were taken from our department driven BA-DADA face database (issued by the senior author), whereas famous faces were retrieved from the internet. In a pre-study, 40 participants from the same population as the participants of the main study had to rate 40 famous faces according to their familiarity on a 1–5 rating scale (1 = *not famous at all*, 5 = *very famous*). The eight highest ranks of familiarity for each gender (eight female, eight male faces) were used for the present study (*M* = 4.6; *SD* = .3). Famous faces included: Angela Merkel and Barack Obama (politicians); Angelina Jolie, Anne Hathaway, Jennifer Aniston, Keira Knightley, Kristen Stewart, Daniel Radcliffe, George Clooney, and Will Smith (actors); Dieter Bohlen, Justin Timberlake, and Michael Jackson (musicians); Heidi Klum (super model), Kate Middleton (royal), and Bastian Schweinsteiger (soccer star). Famous and non-famous faces were neutral with regard to their expression and all in a frontal perspective. Faces were approximately 7 cm high and 6 cm wide (7.3° and 6.2° of visual angle, respectively).

### Timing, design and procedure

Participants were instructed to respond as quickly and as accurately as possible by pressing the green or the red button on the button box with assignment of the buttons being counterbalanced across participants. The experiment started with calibration on the default 9-point grid, which was accepted if there was an angular error of less than 1° for each point tested. The trial schema was similar to the one realised by Carbon et al. [32]. Each trial started with a brief instruction (2,000 ms): “Is the following picture grotesque or not? Please press the green button if the face appears to be grotesque/not grotesque and the red button if the face appears to be not grotesque/grotesque”. After a blank screen (500 ms), a central fixation cross appeared for 200 ms followed by another blank screen (500 ms). Finally, the famous or non-famous face was presented for 1,000 ms and participants could respond during the presentation or during the following blank screen, which was presented until participants pressed one of the two designated buttons. The procedure of one trial can be extracted from Fig 2. After 128 trials, presented in randomized order, participants were asked about the purpose of the experiment. Furthermore, to ensure that the famous faces from the pre-test were actually recognised as being famous for typical participants, they had to rate all the famous faces presented during the experiment according to their familiarity on a 1–5 rating scale (1 = *not famous at all*, 5 = *very famous*). The whole procedure lasted about 45 minutes.

## Results

None of the participants had to be excluded from analysis because of their unfamiliarity with the famous faces. Average ratings for all participants for all famous faces were at 4.48 (*SD* = .47), ensuring generally very high levels of familiarity.

Behavioural and eye movement data of correct trials only (since incorrect trials could confound mean RT scores) were analysed using repeated measures analysis of variance (ANOVA) with the three within-subjects factors of *orientation* (upright/inverted), *Thatcherisation* (yes/no) and *familiarity* (famous/non-famous). Multiple comparisons were Bonferroni-corrected.

### Behavioural results

#### Reaction times (RTs)

First, RT outliers were excluded from further analysis, which were defined as such that RTs are more than two standard deviations apart from the participant’s individual mean reaction time. On average 3.70 (*SD* = 4.72) trials for each participant had to be excluded.

RTs for correct trials only (excluding on average 13.89 (*SD* = 6.74) trials for each participant which corresponds to a rate of 10.85% of all trials) were then further analysed. The ANOVA revealed no effect of *orientation* (upright: *M* = 1,200.00 ms; *SD* = 628.23¸ inverted: *M* = 1,303.55 ms; *SD* = 510.79), *F*(1, 22) < 1; *p* = .380; *n*.*s*., *Thatcherisation* (yes: *M* = 1,327.61 ms; *SD* = 866.65; no: *M* = 1,175.91 ms; *SD* = 272.37), *F*(1, 22) = 1.32; *p* = .263; *n*.*s*. or *familiarity* (famous: *M* = 1,157.71 ms; *SD* = 245.04; non-famous: *M* = 1,345.80 ms; *SD* = 893.98) on RTs, *F*(1, 22) = 2.97; *p* = .099; *n*.*s*. None of the interactions did reach significance.

#### Accuracy

**Fig 3:**
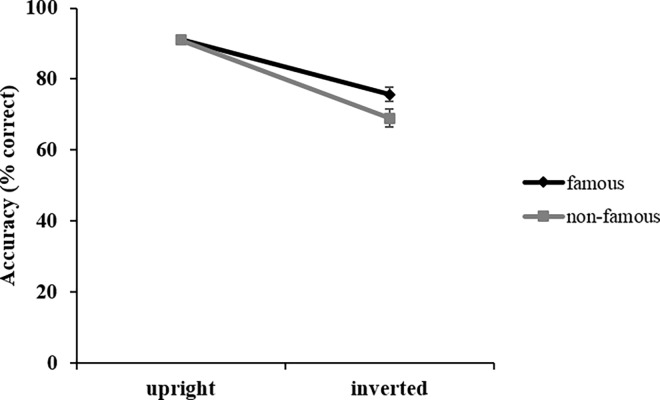
Accuracy data. Interaction between *familiarity* (famous/non-famous) and *orientation* (upright/inverted) in accuracy data. Error bars indicate ± 1 standard error of the mean (SEM).

Correct responses were defined as the assessments of a Thatcherised face as being grotesque and the assessments of a normal face as being not grotesque. The ANOVA revealed a different pattern of results compared to the RT analysis: first, a main effect of *orientation*, *F*(1, 22) = 113.65; *p* < .001; η_p_^2^ = .84. Participants had significantly higher accuracy to upright (*M* = 91.4%; *SD* = 6.1) compared to inverted faces (*M* = 72.3%; *SD* = 19.5). *Thatcherisation* did not have a significant effect on accuracy, *F*(1, 22) < 1; *p* = .77; *n*.*s*. However, *familiarity* had a significant effect on accuracy, *F*(1, 22) = 15.01; *p* = .001; η_p_^2^ = .41, showing that responses to famous faces were significantly more accurate (*M* = 83.8%; *SD* = 10.9) than to non-famous faces (*M* = 80.0%; *SD* = 14.7). The only significant interaction was between *familiarity* and *orientation*, *F*(1, 22) = 5.48; *p* = .029; η_p_^2^ = .20. Fig 3 displays and clarifies the meaning of this interaction. It shows that only if faces were inverted, accuracy was significantly higher for famous (*M* = 75.7%; *SD* = 16.8) compared to non-famous faces (*M* = 69.0%; *SD* = 22.3), *F*(1, 22) = 10.77; *p* = .003; η_p_^2^ = .33. For upright faces, there was no difference between famous (*M* = 91.8%; *SD* = 5.0) compared to non-famous faces (*M* = 91.0%; *SD* = 7.2), *F*(1, 22) < 1; *p* = .834; *n*.*s*.

#### Inverse Efficiency Scores (IES; [43–45])

We used IES for being able to compensate for differences in proportion of errors due to speed-accuracy trade-offs. IES can be seen as the “average energy consumed by the system over time” ([45], p. 6). The usage of IES has one big advantage: to be able to report one single variable, but it should also not be neglected that IES integrate two different kinds of measurements with different distributions; Bruyer and Brysbaert (2011) showed that the IES might be helpful for providing simple data analysis and thus sharp interpretations, but caution is required if some pre-assumptions are not met, e.g. if faster responses come along with higher error rates which indicates a change of the criterion. Following Bruyer and Brysbaert’s (2011, [45]) advice, we therefore will not rely our analyses solely on IES but have also have reported analyses on the basis of the individual measures which the IES subsumes. The higher the IES scores are the worse the performance of participants was.

Regarding the IES, a repeated measures ANOVA revealed a main effect of *orientation*, *F*(1, 22) = 17.36; *p* < .001; η_p_^2^ = .44. Participants showed significantly lower IES to upright (*M* = 1,338.97 ms; *SD* = 828.55) compared to inverted faces (*M* = 2,224.70 ms; *SD* = 1,893.40). *Thatcherisation* did not have a significant effect on IES, *F*(1, 22) = 1.18; *p* = .290; *n*.*s*. *Familiarity* did also not have a significant effect on IES, *F*(1, 22) = 3.73; *p* = .067; *n*.*s*. We did not obtain a significant interaction between *Thatcherisation* and *familiarity*, *F*(1, 22) = 1.33; *p* = .26; *n*.*s*., nor did any other factors interact.

#### Sensitivity (d′)

**Fig 4:**
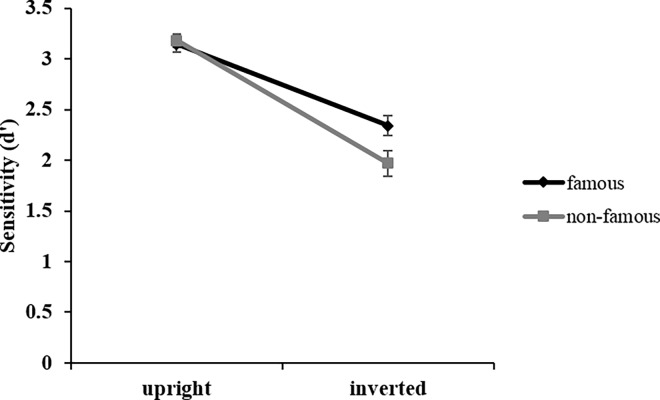
Sensitivity. Interaction between *familiarity* (famous/non-famous) and *orientation* (upright/inverted) in sensitivity data. Error bars indicate ± 1 standard error of the mean (SEM).

In signal detection theory, the sensitivity index d′ refers to how easy or hard it is for participants to detect if the face is Thatcherised (i.e., it looks grotesque) or not. Calculation of d′ is based on the proportion of misses, false alarms, correct rejections, and hits. For correct calculation of d′, hit rates of 1 and false alarm rates of 0 were standard corrected. The ANOVA revealed a main effect of *orientation*, *F*(1, 22) = 159.78; *p* < .001; η_p_^2^ = .88. Participants showed significantly higher sensitivity for evaluating upright (*M* = 3.16; *SD* = .49) compared to inverted faces (*M* = 2.16; *SD* = .88). *Thatcherisation* also had a significant effect on sensitivity, *F*(1, 22) = 11.56; *p* = .03; η_p_^2^ = .34. Participants showed significantly higher sensitivity for evaluating Thatcherised (*M* = 2.97; *SD* = .52) compared to non-Thatcherised faces (*M* = 2.35; *SD* = .85). *Familiarity* had a significant effect on sensitivity as well, *F*(1, 22) = 10.15; *p* = .004; η_p_^2^ = .32, showing that responses to famous faces were significantly more sensitive (*M* = 2.74; *SD* = .59) than to non-famous faces (*M* = 2.57; *SD* = .78). The only significant interaction was between *familiarity* and *orientation*, *F*(1, 22) = 5.87; *p* = .024; η_p_^2^ = .21. Fig 4 displays and clarifies the meaning of this interaction. It shows that only when faces were inverted, sensitivity was significantly higher for famous (*M* = 2.34; *SD* = .75) compared to non-famous faces (*M* = 1.97; *SD* = 1.01), *F*(1, 22) = 11.39; *p* = .003; η_p_^2^ = .34, when faces were upright, there was no difference for famous (*M* = 3.14; *SD* = .43) compared to non-famous faces (*M* = 3.18; *SD* = .55), *F*(1, 22) < 1; *p* = .681; *n*.*s*.

### Eye tracking

Data was analysed using SR Research Data Viewer 1.11.1. An eye position remaining within a 50 pixel area for more than 100 ms was considered as a fixation (e.g., [46]). Fixations shorter than 100 ms were integrated with the immediately preceding or following fixation if that fixation lay within one degree of visual angle, otherwise the fixation was excluded. Such short fixations usually result from false saccade planning and are unlikely to reflect meaningful information processing (see [47]).

#### Number of fixations

**Fig 5:**
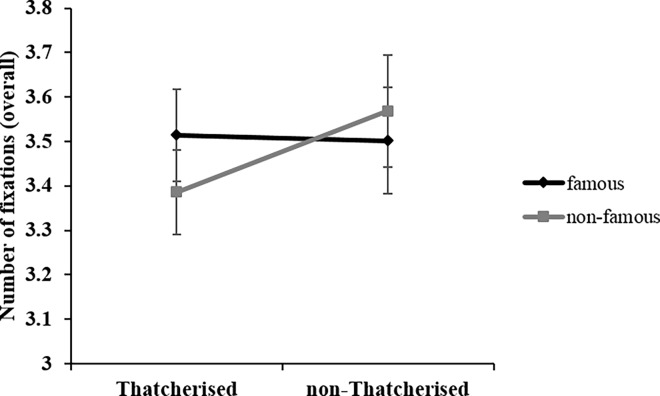
Overall fixation data. Interaction between *familiarity* (famous/non-famous) and *Thatcherisation* (yes/no) in number of fixations data. Error bars indicate ± 1 standard error of the mean (SEM).

The ANOVA revealed a main effect of *orientation*, *F*(1, 22) = 14.24; *p* = .001; η_p_^2^ = .39. Participants made significantly fewer fixations to upright (*M* = 3.38; *SD* = .58) compared to inverted faces (*M* = 3.60; *SD* = .58). *Thatcherisation* did not have a significant effect on the number of fixations, *F*(1, 22) = 1.75; *p* = .20; *n*.*s*. *Familiarity* also did not have a significant effect on the number of fixations, *F*(1,22) < 1; *p* = .48; *n*.*s*. The only significant interaction was between *familiarity* and *Thatcherisation*, *F*(1, 22) = 5.71; *p* = .026; η_p_^2^ = .21. Fig 5 displays and clarifies the meaning of this interaction. It shows that only in non-famous faces, there were fewer fixations for Thatcherised (*M* = 3.39; *SD* = .54) compared to non-Thatcherised faces (*M* = 3.57; *SD* = .64), *F*(1, 22) = 5.74; *p* = .026; η_p_^2^ = .21. For famous faces, there was no difference in fixations for Thatcherised (*M* = 3.39; *SD* = .53) compared to non-Thatcherised faces (*M* = 3.39; *SD* = .61), *F*(1, 22) < 1; *p* = .882; *n*.*s*.

#### Duration of fixations

Regarding duration of fixation, the ANOVA revealed a main effect of *orientation*, *F*(1, 22) = 13.93; *p* = .001; η_p_^2^ = .39. Participants made significantly shorter fixations to upright (*M* = 80.24 ms; *SD* = 65.25) compared to inverted faces (*M* = 117.91 ms; *SD* = 63.18). *Thatcherisation* had a significant effect on duration of fixations, *F*(1, 22) = 11.57; *p* = .003; η_p_^2^ *=* .35. Duration of fixations to Thatcherised faces was shorter (*M* = 90.17 ms; *SD* = 61.88) than to non-Thatcherised faces (*M* = 107.97 ms; *SD* = 66.54). *Familiarity* did not have an effect on the duration of fixations, *F*(1,22) < 1; *p* = .925; *n*.*s*. The factors *familiarity* and *Thatcherisation* interacted significantly, *F*(1, 22) = 5.20; *p* = .033; η_p_^2^ = .19. Only for non-famous faces were there significantly longer fixations for non-Thatcherised (*M* = 112.14 ms; *SD* = 66.53) compared to Thatcherised faces (*M* = 85.60 ms; *SD* = 67.46), *F*(1, 22) = 16.60; *p* = .001; η_p_^2^ = .43. For famous faces, there was no difference between non-Thatcherised (*M* = 103.80 ms; *SD* = 66.55) and Thatcherised faces (*M* = 94.75 ms; *SD* = 56.31), *F*(1, 22) = 1.97; *p* = .175; *n*.*s*. There was also a significant interaction between *Thatcherisation* and *orientation*, *F*(1, 22) = 9.18; *p* = .006; η_p_^2^ = .29, showing only for inverted faces significantly longer fixations for non-Thatcherised (*M* = 131.93 ms; *SD* = 65.01) compared to Thatcherised faces (*M* = 103.89 ms; *SD* = 61.34), *F*(1, 22) = 21.59; *p* < .001; η_p_^2^ = .50. For upright faces, there was no difference between non-Thatcherised (*M* = 84.01 ms; *SD* = 68.07) and Thatcherised faces (*M* = 76.46 ms; *SD* = 62.42), *F*(1, 22) = 1.39; *p* = .252; *n*.*s*.

#### Region of interest: mouth

**Fig 6:**
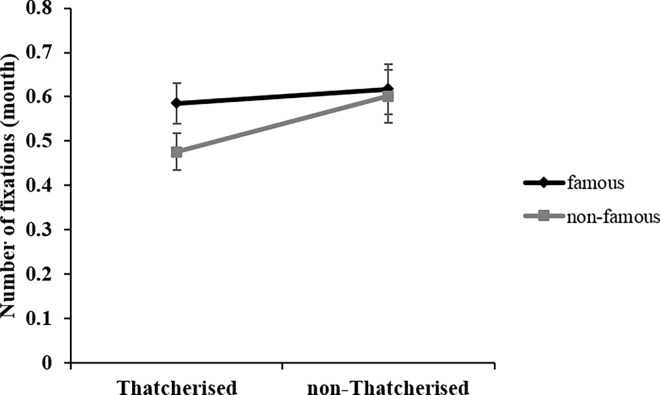
Mouth region fixation data. Interaction between *familiarity* (famous/non-famous) and *Thatcherisation* (yes/no) in number of fixations data. Error bars indicate ± 1 standard error of the mean (SEM).

Regarding number of fixations in the mouth region, the ANOVA revealed a main effect of *orientation*, *F*(1, 22) = 8.99; *p* = .007; η_p_^2^ = .29. Participants made significantly fewer fixations to upright (*M* = .40; *SD* = .36) compared to inverted faces (*M* = .74; *SD* = .40). *Thatcherisation* had a significant effect on number of fixations, *F*(1, 22) = 4.58; *p* = .044; η_p_^2^ *=* .17. Number of fixations to Thatcherised faces was lower (*M* = .53; *SD* = .34) than to non-Thatcherised faces (*M* = .61; *SD* = .42). *Familiarity* also had a significant effect on the number of fixations, *F*(1,22) = 5.45; *p* = .029; η_p_^2^ = .20. The number of fixations to famous faces was higher (*M* = .60; *SD* = .39) than to non-famous faces (*M* = .54; *SD* = .37). The factors *familiarity* and *Thatcherisation* interacted significantly, *F*(1, 22) = 4.61; *p* = .043; η_p_^2^ = .17. Fig 6 displays and clarifies the meaning of this interaction. It shows that only in Thatcherised faces, there were significantly more fixations for famous (*M* = .59; *SD* = .36) compared to non-famous faces (*M* = .48; *SD* = .32), *F*(1, 22) = 15.57; *p* = .001; η_p_^2^ = .41, and only for non-famous faces significantly more fixations for non-Thatcherised (*M* = .60; *SD* = .41) compared to Thatcherised faces (*M* = .48; *SD* = .32), *F*(1, 22) = 11.76; *p* = .002; η_p_^2^ = .35. There was also a significant interaction between *Thatcherisation* and *orientation*, *F*(1, 22) = 6.63; *p* = .017; η_p_^2^ = .23, showing only for inverted faces significantly more fixations for non-Thatcherised (*M* = .82; *SD* = .45) compared to Thatcherised faces (*M* = .66; *SD* = .36), *F*(1, 22) = 8.25; *p* = .009; η_p_^2^ = .27. For upright faces, there was no difference between non-Thatcherised (*M* = .40; *SD* = .40) and Thatcherised faces (*M* = .40; *SD* = .32), *F*(1, 22) < 1; *p* = .969; *n*.*s*.

Regarding the duration of fixation in the mouth region, the ANOVA revealed a main effect of *orientation*, *F*(1, 22) = 8.18; *p* = .009; η_p_^2^ = .27. Participants made significantly shorter fixations to upright (*M* = 98.07 ms; *SD* = 92.29) compared to inverted faces (*M* = 172.94 ms; *SD* = 97.48). *Thatcherisation* had a significant effect on the duration of fixations, *F*(1, 22) = 10.93; *p* = .003; η_p_^2^ *=* .33. The duration of fixations to Thatcherised faces was shorter (*M* = 122.01 ms; *SD* = 87.88) than non-Thatcherised faces (*M* = 148.99 ms; *SD* = 101.88). *Familiarity* did not have a significant effect on the duration of fixations, *F*(1,22) = 2.35, *p* = .139; *n*.*s*. The factors *familiarity* and *Thatcherisation* interacted significantly, *F*(1, 22) = 6.69; *p* = .017; η_p_^2^ = .23. Only in Thatcherised faces were there significantly longer fixations for famous (*M* = 135.88 ms; *SD* = 90.22) compared to non-famous faces (*M* = 108.14 ms; *SD* = 85.55), *F*(1, 22) = 8.87; *p* = .007;, η_p_^2^ = .29, and only for non-famous faces significantly longer fixations for non-Thatcherised (*M* = 151.82 ms; *SD* = 98.93) compared to Thatcherised faces (*M* = 108.14 ms; *SD* = 85.55), *F*(1, 22) = 23.22; *p* < .001; η_p_^2^ = .51. There was also a significant interaction between *Thatcherisation* and *orientation*, *F*(1, 22) = 19.49; *p* < .001; η_p_^2^ = .47, showing significantly longer fixations for inverted faces for non-Thatcherised (*M* = 198.72 ms; *SD* = 106.01) compared to Thatcherised faces (*M* = 147.16 ms; *SD* = 88.95), *F*(1, 22) = 24.84; *p* < .001; η_p_^2^ = .53. For upright faces, there was no difference between non-Thatcherised (*M* = 99.26 ms; *SD* = 97.75) and Thatcherised faces (*M* = 96.87 ms; *SD* = 86.82), *F*(1, 22) < 1; *p* = .801; *n*.*s*.

Regarding dwell time (sum of durations from all fixations and saccades that hit the region of interest) in the mouth region, the ANOVA revealed a main effect of *orientation*, *F*(1, 22) = 10.74; *p* = .003, η_p_^2^ = .33. Dwell time was significantly shorter to upright (*M* = 106.58; *SD* = 102.92) compared to inverted faces (*M* = 216.55; *SD* = 131.23). *Thatcherisation* had a significant effect on the number of fixations, *F*(1, 22) = 15.75; *p* = .001; η_p_^2^ *=* .42. Dwell time to Thatcherised faces was shorter (*M* = 140.59 ms; *SD* = 105.41) than to non-Thatcherised faces (*M* = 182.55 ms; *SD* = 128.74). *Familiarity* also had a significant effect on dwell time, *F*(1,22) = 4.81, *p* = .039; η_p_^2^ = .18. Dwell time to famous faces was longer (*M* = 171.37 ms; *SD* = 123.04) than to non-famous faces (*M* = 151.76 ms; *SD* = 111.10). The factors *familiarity* and *Thatcherisation* interacted significantly, *F*(1, 22) = 11.06; *p* = .003; η_p_^2^ = .33. Only in Thatcherised faces were there significantly longer dwell times for famous (*M* = 160.82 ms; *SD* = 112.14) compared to non-famous faces (*M* = 120.35 ms; *SD* = 98.68), *F*(1, 22) = 17.60; *p* < .001; η_p_^2^ = .44, and only for non-famous faces, significantly longer dwell times for non-Thatcherised (*M* = 183.16 ms; *SD* = 123.53) compared to Thatcherised faces (*M* = 120.35 ms; *SD* = 98.68), *F*(1, 22) = 35.53; *p* < .001; η_p_^2^ = .62. There was also a significant interaction between *Thatcherisation* and *Orientation*, *F*(1, 22) = 20.57; *p* < .001; η_p_^2^ = .48, only for inverted faces, with a significantly longer dwell time for non-Thatcherised (*M* = 255.10 ms; *SD* = 146.21) compared to Thatcherised faces (*M* = 178.00 ms; *SD* = 116.25), *F*(1, 22) = 27.51; *p* < .001; η_p_^2^ = .56. For upright faces, there was no difference between non-Thatcherised (*M* = 109.99 ms; *SD* = 111.27) and Thatcherised faces (*M* = 103.17 ms; *SD* = 94.56), *F*(1, 22) < 1; *p* = .552; *n*.*s*.

#### Region of interest: eyes

Regarding number of fixations in the eye region, the ANOVA revealed no main effect of *orientation*, *F*(1, 22) = 1.86; *p* = .186; *n*.*s*. *Thatcherisation* also had no effect on the number of fixations, *F*(1, 22) < 1; *p* = .412; *n*.*s*. *Familiarity*, however, had a significant effect on the number of fixations, *F*(1,22) = 33.39; *p* < .001; η_p_^2^ = .60. The number of fixations to famous faces was lower (*M* = 1.92; *SD* = .69) than to non-famous faces (*M* = 2.22; *SD* = .66). There was a significant interaction between *Thatcherisation* and *orientation*, *F*(1, 22) = 5.19; *p* = .033; η_p_^2^ = .19, showing only for upright faces significantly more fixations for non-Thatcherised faces (*M* = 2.27; *SD* = .66) compared to Thatcherised faces (*M* = 2.10; *SD* = .53), *F*(1, 22) = 4.68; *p* = 042; η_p_^2^ = .18. For inverted faces, there was no difference between non-Thatcherised (*M* = 1.91; *SD* = .77) and Thatcherised faces (*M* = 1.99; *SD* = .73), *F*(1, 22) = 1.10; *p* = .305; *n*.*s*.

Regarding duration of fixation in the eye region, the ANOVA revealed no effect of *orientation*, *F*(1, 22) < 1; *p* = .958; *n*.*s*. or *Thatcherisation* on the duration of fixations, *F*(1, 22) = 3.02; *p* = .096; *n*.*s*. *Familiarity* had a significant effect on the duration of fixations, *F*(1,22) = 6.89; *p* = .015; η_p_^2^ = .24. Duration of fixations to non-famous faces was higher (*M* = 67.75 ms; *SD* = 97.22) than to famous faces (*M* = 57.53 ms; *SD* = 99.21). None of the factors interacted significantly.

Regarding dwell time in the eye region, the ANOVA revealed no effect of *orientation*, *F*(1, 22) = 1.51; *p* = .232; *n*.*s*. or *Thatcherisation*, *F*(1, 22) < 1; *p* = .851; *n*.*s*. *Familiarity* had a significant effect on the dwell time, *F*(1,22) = 23.58; *p* < .001; η_p_^2^ = .52. Dwell time to non-famous faces was longer (*M* = 631.05 ms; *SD* = 208.17) than to famous faces (*M* = 542.38 ms; *SD* = 218.02). There was a significant interaction between *Thatcherisation* and *orientation*, *F*(1, 22) = 18.06; *p* < .001; η_p_^2^ = .45, showing only for non-Thatcherised faces significantly longer dwell times for upright (*M* = 643.10 ms; *SD* = 221.16) compared to inverted faces (*M* = 527.80 ms; *SD* = 203.31), *F*(1, 22) = 4.30; *p* = .050; η_p_^2^ = .164. For Thatcherised faces, there was no difference between upright (*M* = 594.98 ms; *SD* = 206.66) and inverted faces (*M* = 580.98 ms; *SD* = 221.24), *F*(1, 22) < 1; *p* = .791; *n*.*s*.

### Summary results

Regarding potential differences in processing famous and non-famous faces, behavioural data showed higher accuracy and higher sensitivity for famous compared to non-famous faces, but only if faces were inverted. We did not find differences in RTs.

Overall eye tracking data revealed a lower number and a shorter duration of fixations only for non-famous faces for Thatcherised compared to non-Thatcherised faces. Looking at the areas of interest–eye and mouth regions–results showed for the eye region a lower number, shorter duration and dwell time of fixations for famous compared to non-famous faces. In the mouth region, number, duration and dwell time of fixations was higher for famous compared to non-famous faces, but only if they were Thatcherised. Furthermore, only for non-famous faces, we found a higher number, duration and dwell time of fixations for non-Thatcherised faces compared to Thatcherised.

## Discussion

We tested whether familiarity modulates key variables for the processing of faces, which we varied with respect to orientation (upright vs. inverted) and Thatcherisation (Thatcherised vs. non-Thatcherised). On a behavioural level, we analysed RTs and correctness of grotesqueness assessments (accuracy), plus an integrative measure known as Inverse Efficiency Score (IES). Additionally, we analysed the eye movement patterns while inspecting the faces, focusing on duration and the number of fixations on cardinal face regions such as eyes and mouth.

We could reveal higher accuracy and sensitivity for famous compared to non-famous faces, but only when the faces were inverted. RTs as well as IES scores were not affected by familiarity. Eye tracking data revealed a lower number and a shorter duration of fixations only for non-famous faces, for Thatcherised compared to non-Thatcherised faces. Thatcherisation did not have an impact on fixations for famous faces, but we detected dissociate scanning behaviours between famous vs. non-famous faces. Whereas we uncovered a lower number, shorter duration and dwell time of fixations for famous compared to non-famous faces in the eye region, scanning in the mouth region showed a contrasting pattern (i.e., number, duration and dwell time of fixations was higher for famous compared to non-famous faces–but this was only the case if they were Thatcherised).

Expertise–which we have for famous faces–should increase configural processing and therefore result in faster and more accurate processing. Several studies found faster and more accurate recognition of familiar faces (e.g., [22–24]). Our participants, however, were not faster in processing familiar (i.e., famous) faces. Several reasons might be responsible for this different pattern of results: first of all, we used famous faces instead of just familiar faces; secondly, our task did not involve recognising faces, but just deciding the grotesqueness of the presented face. With this given task, participants were, however, more accurate in deciding if famous faces were grotesque or not when they were inverted, probably due to better knowledge of what the people look like when presented normally. In accord with several studies (e.g., [4; 38; 39]), we expected no influence of familiarity on the perception (measured in reaction times) of the Thatcher Illusion through familiarity, which the missing interaction of familiarity and Thatcherisation in our data actually showed.

Barton et al. (2006) showed faster scanning for upright famous faces in general [19 & 20]. Overall shorter fixations, dwell time, and a lower number of fixations for upright famous faces was therefore expected. Heisz and Shore (2008) showed that participants focussed with increasing familiarity of the presented faces on the eyes rather than on any other region ([31]; similar to [19]) and interpreted their findings as an optimisation routine, but also a smaller focus on the eyes region of famous faces. Barton et al. (2006) however, showed a smaller focus on the eye region of famous faces. Therefore, both results could have been predicted. We found a shorter duration of fixations only for non-famous faces for Thatcherised as compared to non-Thatcherised faces, and significantly less and shorter fixations in the eye region for famous compared to non-famous faces, but not in the mouth region. Instead, there were more and longer fixations in the mouth region for famous compared to non-famous faces, but only when faces were Thatcherised. Our results are more in line with the findings by Barton et al. (2006, [19]), who found, in a face recognition task, that more fixations were made in the eyes and upper face region in novel compared to famous faces; however, their faces were not Thatcherised. One reason might be that areas highly indicative for the identification of faces, foremost the eyes, are not processed in depth for famous faces due to the fast and easy recognition process, i.e., less need for scanning the eyes of famous faces compared to novel faces.

Since accuracy was better for processing famous faces, results might argue at first sight in favour of a more complex process, e.g., by proposing additional processes. It might, in contrast, also indicate less efficient but still reliable processing due to re-processing the faces in an alternative way as configural processing is not efficiently available. Carbon et al. (2007, [32]) tested processing of the Thatcher Illusion in persons suffering from congenital prosopagnosia, who are susceptible to being unable to efficiently process faces in a configural way (e.g., [48–51]). Specifically, the participants had to assess the grotesqueness of Thatcherised vs. non-Thatcherised faces when rotated from an upright to an inverted orientation in steps of 30 (degrees). RTs in relation to orientation showed dissociate patterns for prosopagnosic individuals and matched controls. Depending on the rotation, RTs of the controls followed a strong sigmoid function, whereas RTs of the prosopagnosic individuals approached a linear function. The authors interpreted their findings as an impaired configural processing as the cause of the lack of “face expertise” in prosopagnosia [32]. It seems that non-famous faces in the present study were processed in a similar way as prosopagnosics process faces in general, as such faces are processed in a much more inefficient way than well-known, famous faces due to an absence of expertise for such unfamiliar faces.

Regarding our eye tracking data, significantly more fixations were made in Thatcherised famous faces compared to Thatcherised non-famous faces. Only for non-famous faces, participants made significantly more fixations for non-Thatcherised faces compared to Thatcherised faces. In the previous section, we reported that behavioural results (RT and accuracy) indicated that inversion instead of Thatcherisation caused differences in the perception of famous vs. non-famous faces. The fixation behaviour, in contrast, revealed that Thatcherisation seems more disruptive to the observer in famous faces than in non-famous faces, which reflected the need for more fixations until participants could decide—although the decision was then a more accurate one, as shown before. Again, this indicates that famous faces are processed in a more elaborate, more expertise-based way than non-famous faces. Such findings only partially mirror the evidence collated in a seminal review paper on unfamiliar face processing by Hancock and colleagues, who stated that “subjects perform surprisingly poorly” ([52], p.335) when they have to process *unfamiliar* faces (see also [53; 54]). Our results reflect this statement for the inverted condition and furthermore, demonstrate that existing theories are not fully sufficient to predict the full pattern of effects in a concordant way; therefore, advancement of such theories, especially addressing the efficient but also fragile nature of *configural processing*, seems mandatory. Altogether, famous faces seem to be processed in a more elaborate, more expertise-based way than non-famous faces, whereas non-famous, inverted faces seem to cause difficulties in accurate and sensitive processing.

## Supporting Information

S1 Dataset Full data set including behavioural and eye movement data. (DAT)
